# Inter-limb asymmetry in postural control: Role of individual and contextual factors

**DOI:** 10.1055/a-2716-5569

**Published:** 2025-11-18

**Authors:** Thomas Muehlbauer, Katharina Borgmann, Sam Limpach, Dirk Krombholz, Stefan Panzer

**Affiliations:** 1 Division of Movement and Training Sciences/Biomechanics of Sport, Universitat Duisburg-Essen, Essen, Germany; 2 Institute of Biomechanics and Orthopaedics, German Sport University Cologne, Cologne, Germany; 3 Institute of Sport Science, Saarland University, Saarbrücken, Germany; 4 Nachwuchsakademie, Dynamo Dresden, Dresden, Germany; 5 Department of Health and Kinesiology, Texas A&M University, College Station, United States

**Keywords:** balance, inter-limb difference, side-to-side difference, athletes, task Specificity

## Abstract

Little is known about how individual and contextual factors affect inter-limb differences in balance performance. Thus, we investigated how these factors influence inter-limb asymmetry in balance. Sixty-four soccer players with diverging levels of training experience (i. e., 2–5 or 6–9 years), 73 swimmers, and 60 age-matched non-athletes performed balance tests with different task specificity (i. e., ecological vs *.* non-ecological). The magnitude of inter-limb differences was quantified by calculating the limb symmetry index (LSI). Inter-limb performance differences were significantly ( *p* =0.012) lower in athletes with (i. e., soccer players) than without (i. e., swimmers) the preferential use of one leg for postural control. However, differences between limbs did not significantly differ among players with diverging levels of training experience. Further, the observed inter-limb differences in soccer players emerged during ecological test conditions only. Our results suggest that the predominant use of one limb compared to the other for balance requirements does not necessarily lead to a large magnitude of inter-limb asymmetry in soccer players and is also not significantly influenced by the level of training experience. However, from a practitioners’ perspective, ecological as opposed to non-ecological test conditions seem to be more suitable for detecting inter-limb asymmetry in soccer players.

## Introduction

Inter-limb asymmetry or side-to-side performance difference between corresponding extremities emerge as a consequence of an athlete’s long-standing preferential use of one limb to perform athletic tasks in their sport 1 . Theoretical and empirical support for the development of an inter-limb asymmetry is drawn largely from research investigating the effects of side differences and athletic performance 1 2 3 4 . The magnitude of inter-limb asymmetry can be quantified by calculating performance differences between both extremities from unilateral tests 5 . In soccer, small differences in inter-limb asymmetry provide an increase in performance as seen in higher ball speeds when kicking with the dominant compared to the non-dominant leg 6 . However, there is evidence 3 that lower limb asymmetry magnitudes surpassing 10–15% could have a negative impact on athletic performance (e. g., change-of-direction speed, sprint time, jump height). In addition, previous research 7 8 in athletes reported that exceeding a reach asymmetry value of 4 cm during the assessment of dynamic balance is associated with a higher injury risk for the lower extremities.

Against this background, the investigation of inter-limb asymmetry appears to be relevant from both a performance and health perspective. Regarding postural control, there are numerous studies 9 10 11 12 that have already examined the prevalence and magnitude of inter-limb performance differences. However, varying findings were reported 13 that may be attributed to discrepancies in the applied methodology (e. g., type of sport, competition level, balance assessment). In addition, previous studies did not distinguish between individual and contextual factors which are known to influence postural control 14 .

With regard to individual factors (i. e., practiced type of sport; experience level), it has been suggested that athletes who participate in a sport with the preferential use of one leg for postural control (e. g., soccer players) will show greater inter-limb asymmetry than athletes who participate in a sport with no preferential use of one leg for balance requirements (e. g., swimmers) 13 15 . In addition and with regard to soccer players, there is empirical support 9 that inter-limb asymmetry increases the higher the experience level and the longer the associated experience in the preferential use of one leg for postural control is.

Regarding contextual factors (i. e., level of task specificity), Paillard 16 distinguishes between ecological (i. e., specific postural conditions related to the practiced type of sport) and non-ecological (i. e., decontextualized postural control conditions in relation to the practiced type of sport) test conditions. Consequently, it is logical to assume that soccer players would show inter-limb asymmetry in postural control, especially under ecological test conditions (e. g., single leg landing/standing).

In sum, the existing studies on inter-limb asymmetry in postural control do not differentiate between individual and contextual factors 9 10 11 12 . Due to this lack of knowledge, the present study aimed to investigate the role of these factors on inter-limb performance differences in postural control. We hypothesised that side-to-side differences would 1) be larger in athletes with (i. e., soccer players) than without (i. e., swimmers) the preferential use of one leg for postural control and age-matched non-athletes; 2) be more pronounced in soccer players with a high (i. e., 6–9 years) compared to a low (i. e., 2–5 years) level of training experience; and 3) emerge in soccer players when tested under ecological compared to non-ecological conditions.

## Material and Methods

### Participants and sample size estimation

In the present study, athletes with (i. e., soccer players) and without (i. e., swimmers) the preferential use of one leg for postural control and different levels of training experience as well as age-matched non-athletes were enrolled. Based on the criteria for defining athletes’ experience level provided by Swann et al. 17 , we differentiated between soccer players with 2–5 years or 6–9 years of training experience at the athlete’s highest level. An a priori power analysis using G*Power 18 with the following input parameters was performed: effect size ( *f* =0.25), type I error (α=0.05), type II error ( *1-β* =0.80), number of groups ( *n* =3), number of measurements ( *n* =4), and correlation between measurements ( *r* =0.80) 19 . The analysis revealed that a total sample size of *N=* 135 participants would be sufficient to find significant differences that will be large enough to be considered worthwhile. One-hundred ninety-seven subjects participated in this cross-sectional study after experimental procedures were explained ( Table 1 ). Precisely, 64 soccer players ( *n* =20 females, age: 14.0±1.8 years, years from peak height velocity [PHV]: –0.55±1.33, body height: 166.6±11.3 cm, body mass: 57.3±12.5 kg), 73 swimmers ( *n* =40 females, age: 13.8±2.7 years, years from PHV: –0.32±2.07, body height: 165.8±13.9 cm, body mass: 56.8±14.6 kg), and 60 non-athletes ( *n* =33 females, age: 14.1±1.1 years, years from PHV: –0.40±1.04, body height: 165.2±10.6 cm, body mass: 61.5±15.9 kg) were enrolled in the present study. All participants were free of any musculoskeletal dysfunction, neurological impairment, or orthopedic pathology within the preceding three months. Participant’s assent and written informed consent of the parents or legal guardians were obtained before the start of the study. The Human Ethics Committee at an institution affiliated with one of the authors approved the study protocol (approval number: TM_04.06.2020) 20 .

**Table:** **Table 1** Characteristics of the participants ( *N* =197).

Characteristic	Soccer players	Swimmers	Non-athletes	*p* -value
Sample size ( *N* )	64	73	60	–
Gender (females; *n* )	20	40	33	–
Age (years)	14.0±1.8	13.8±2.7	14.1±1.1	0.779
Maturity offset (years from PHV)*	– 0.55±1.33	– 0.32±2.07	– 0.40±1.04	0.706
Body height (cm)	166.6±11.3	165.8±13.9	165.2±10.6	0.802
Body mass (kg)	57.3±12.5	56.8±14.6	61.5±15.9	0.136
Training experience (2–5 / 6–9 years, *n* )**	29 / 35	–	–	–

*Note:* Values are means±standard deviations. *Maturity offset was calculated as years from peak height velocity by using the formula provided by Mirwald et al. 20 . **in accordance with Swann et al. 17 , we differentiated between soccer players with 2–5 years or 6–9 years of training experience at the athlete’s highest level. PHV, peak height velocity.

### Experimental procedure

**Fig. 1:**
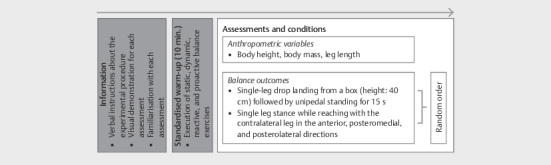
Schematic diagram of the applied experimental procedure.

Upon entering the laboratory, the participants received standardized verbal instructions regarding the experimental procedure with a visual demonstration and familiarization of all assessments. Subsequently, the following schedule was followed: 1) assessments of anthropometric variables (i. e., body height, body mass, leg length); 2) execution of a standardized 10-minute warm-up program consisting of static (e. g., unipedal stance), dynamic (e. g., beam walking), reactive (e. g., jump landings), and proactive (e. g., maximal forward/backward leaning) balance exercises; and 3) assessment of balance performance in a random order ( Fig. 1 ). All assessments were conducted by the same investigators (i. e., degreed sport scientists).

### Assessments of anthropometric variables

Body height was determined without shoes to the nearest 0.5 cm with a stadiometer (seca 217, Basel, Switzerland). Further, body mass was measured in light clothing and without shoes to the nearest 100 g with an electronic scale (seca 803). Moreover, the length of the left and right leg was determined by measuring the distance (in cm) from the anterior superior iliac spine to the most distal aspect of the medial malleolus with the participant lying supine 21 .

### Assessments of balance performance

Balance was assessed by using the single-leg drop landing task. In this regard, the participants were asked to stand with one leg on a box (height: 40 cm), drop down and land with the opposite leg. After landing on a balance pad (Airex AG, Sins, Switzerland) that was placed on top of the force plate, the participants’ task was to stand as still as possible for 15 s with hands akimbo. Two practice and three data-collection trials were performed, and the mean was used for further analyses. A trial was discarded and recollected if participants (a) performed a jump rather than a drop landing, (b) lost their balance (i. e., touched the ground with the non-stance leg), or (c) removed the hands from the hips. Validity as well as reliability of the single-leg drop landing task has been shown in previous studies 22 23 .

In addition, the Y Balance Test – Lower Quarter (YBT–LQ) was applied using the Y Balance Test Kit (Functional Movement Systems, Chatham, VA, USA). The kit consists of a centralized stance platform and three pipes that are connected with the platform. The three pipes represent the anterior (AT), posteromedial (PM), and posterolateral (PL) reach directions and are marked in 1.0 cm increments for measurement purposes. All pipes were equipped with a moveable reach indicator. Participants had to stand with one leg on the centralized platform and were instructed to reach with the other leg as far as they could in the AT, PM, and PL directions while maintaining balance. Each participant was asked to perform three practice trials followed by three data-collection trials per leg. Starting with the AT reach direction, this protocol was replicated for the PM and PL directions. A trial was classified as invalid if the participants (a) lost their balance (i. e., step with the reach leg on the ground), (b) lifted the stance leg from the stance platform, (c) stepped on top of the reach indicator for support, or (d) kicked the reach indicator 21 . If an invalid trial occurred, the data was discarded, and the trial was repeated until a total of three valid trials was achieved. The best trial (i. e., absolute maximal reach distance in cm) per leg and reach direction was used for further analyses. The YBT–LQ is a valid and reliable tool to assess balance performance 21 24 .

Based on the distinction made by Paillard 16 , the single-leg drop landing task represents an ecological test condition (i. e., contextualized, specific conditions related to the practiced type of sport) for soccer payers, which repeatedly occurs in training and competition when, for example, performing headers. In contrast, the YBT–LQ corresponds to a non-ecological test condition (i. e., decontextualized, unspecific conditions not related to the practiced type of sport), as unipedal reaching movements do not occur in soccer.

### Data analyses

Regarding the single-leg drop landing task, ground reaction force data (AMTI AccuSway optimized, Watertown, MA, USA) were sampled at 1,000 Hz and low-pass filtered (cut-off frequency: 10 Hz) with a second-order Butterworth filter using a script programmed with MATLAB Version 2023a (The MathWorks, Natick, MA, USA). Afterwards, the frequently used time-to-stabilization outcome measure was calculated, i. e., the time it takes for an individual to return to a stable state following single-leg drop landings 22 . While a variety of calculation methods exist, we decided to use the sequential average (SA) as this is the most reliable method for both the anteroposterior (AP) and mediolateral (ML) directions 23 .

With regard to the YBT–LQ, the normalized (% leg length [LL]) maximal reach distance per reach direction and leg was calculated by dividing the absolute maximal reach distance (in cm) by LL (in cm) and then multiplying by 100. Further, the normalized (% LL) composite score was computed for each leg as the sum of the three maximal reach distances (in cm) per reach direction divided by three times LL (cm) and then multiplied by 100.

Concerning inter-limb performance differences, balance outcomes were used to calculate the limb symmetry index (LSI) according to the formula provided by Bishop et al. 5 : LSI=(1 – non-dominant leg / dominant leg)*100. An LSI<10% is indicative of a normal inter-limb difference and a value above that cut-off indicates inter-limb asymmetry in postural control 25 .

### Statistical analyses

Descriptive data are reported as group mean values and standard deviations (SD) after normal distribution (Shapiro–Wilk test) was examined. A series of univariate analysis of variance (ANOVA) were calculated to detect differences between i) groups (soccer players vs *.* swimmers vs *.* non-athletes) and (ii) soccer players with diverging levels of training experience (2–5 *vs.* 6–9 years). Post-hoc analyses using Bonferroni-adjusted *α* were applied to locate differences between groups. Effect sizes are reported as partial eta-squared value ( *η*_p_^2^ ) and interpreted as small (0.02≤ *η*_p_^2^ ≤0.12), medium (0.13≤ *η*_p_^2^ ≤0.25), or large ( *η*_p_^2^ ≥0.26) 19 . The significance level was a priori set at *p* <0.05 for all tests. All analyses were performed using the Statistical Package for Social Sciences (SPSS) version 28.0 (IBM Corp., Armonk, NY, USA).

## Results

### Comparison of inter-limb performance differences by group and task specificity

**Fig. 2:**
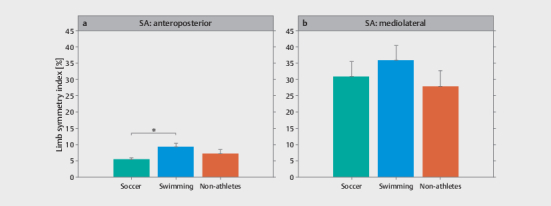
Descriptive statistics for the limb symmetry index obtained from the single-leg drop landing task for the ( **a** ) anteroposterior and ( **b** ) mediolateral direction by group. *Represents a statistically significant difference between groups ( *p<* .05). SA, sequential average.

**Fig. 3:**
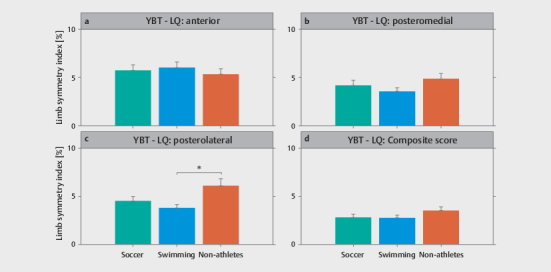
Descriptive statistics for the limb symmetry index obtained from the Y Balance Test – Lower Quarter for the ( **a** ) anterior reach, ( **b** ) posteromedial reach, ( **c** ) posterolateral reach, and ( **d** ) composite score by group. *Represents a statistically significant difference between groups ( *p<* 0.05). YBT–LQ, Y Balance Test – Lower Quarter.

Descriptive statistics for the LSI obtained from the single-leg drop landing task and the YBT–LQ by group are shown in Fig. 2 **a, b** , 3 **a–d** , respectively. Table 2 shows the MANOVA results for differences between groups by balance outcome. For the single-leg drop landing task, there was a significant difference for the LSI in AP direction but not in ML direction. The post-hoc analysis yielded a significantly lower LSI for the soccer players ( *p* =0.012) compared to the swimmers ( Fig. 2a ). For the YBT–LQ, there was a significant group difference for the LSI in the PL reach direction. The post-hoc analysis revealed a significantly lower LSI for the swimmers ( *p* =0.004) compared to the non-athletes ( Fig. 3c ).

**Table:** **Table 2** MANOVA results for differences between groups (soccer players vs *.* swimmers vs *.* non-athletes) by balance outcome.

Outcome	*F* -value	*p* -value ( *η*_p_^2^ )
*Single-leg drop landing task*		
LSI: SA-AP [%]	4.257	**0.016 (0.05)**
LSI: SA-ML [%]	0.767	0.466 (0.01)
*YBT–LQ*		
LSI: AT reach [%]	0.260	0.772 (0.00)
LSI: PM reach [%]	1.780	0.171 (0.02)
LSI: PL reach [%]	5.323	**0.006 (0.05)**
LSI: CS [%]	1.854	0.159 (0.02)

*Note:* Effect size ( *η*_p_^2^ ) was interpreted as small (0.02≤ *η*_p_^2^ ≤0.12), medium (0.13≤ *η*_p_^2^ ≤0.25), or large ( *η*_p_^2^ ≥0.26). Bold values indicate statistically significant group differences. AP, anteroposterior; AT, anterior; CS, composite score; LSI, limb symmetry index; ML, mediolateral; PL, posterolateral; PM, posteromedial; SA, sequential average; YBT–LQ, Y Balance Test – Lower Quarter.

### Comparison of inter-limb performance differences by experience level

**Fig. 4:**
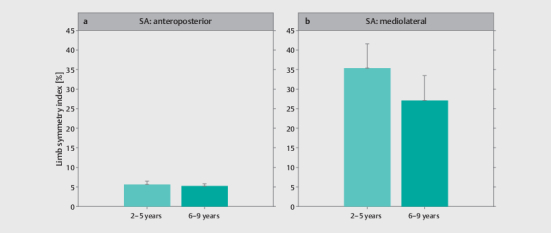
Descriptive statistics for the limb symmetry index obtained from the single-leg drop landing task for ( **a** ) the anteroposterior and ( **b** ) mediolateral direction by soccer players’ experience level. SA, sequential average.

**Fig. 5:**
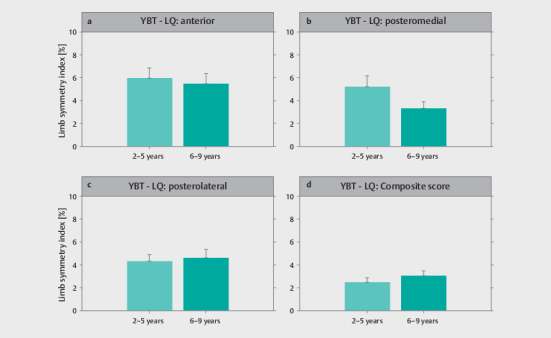
Descriptive statistics for the limb symmetry index obtained from the Y Balance Test – Lower Quarter for the ( **a** ) anterior reach, ( **b** ) posteromedial reach, ( **c** ) posterolateral reach, and ( **d** ) composite score by soccer players’ experience level. YBT–LQ, Y Balance Test – Lower Quarter.

Descriptive statistics for the LSI obtained from the single-leg drop landing task and the YBT–LQ by experience level are displayed in Fig. 4a, b and Fig. 5a–d , respectively. Table 3 represents the MANOVA results for differences between experience levels. Irrespective of task, there were no significant differences between soccer players with different levels of training experience.

**Table:** **Table 3** MANOVA results for differences between soccer players with diverging levels of training experience (2–5 vs *.* 6–9 years) by balance outcome.

Outcome	*F* -value	*p* -value ( *η*_p_^2^ )
*Single-leg drop landing task*		
LSI: SA-AP [%]	0.149	0.701 (0.00)
LSI: SA-ML [%]	0.846	0.362 (0.02)
*YBT–LQ*		
LSI: AT reach [%]	0.158	0.693 (0.00)
LSI: PM reach [%]	3.102	0.083 (0.05)
LSI: PL reach [%]	0.105	0.747 (0.00)
LSI: CS [%]	0.857	0.358 (0.01)

*Note:* Effect size ( *η*_p_^2^ ) was interpreted as small (0.02≤ *η*_p_^2^ ≤0.12), medium (0.13≤ *η*_p_^2^ ≤0.25), or large ( *η*_p_^2^ ≥0.26). AP, anteroposterior; AT, anterior; CS, composite score; LSI, limb symmetry index; ML, mediolateral; PL, posterolateral; PM, posteromedial; SA, sequential average; YBT–LQ, Y Balance Test – Lower Quarter.

## Discussion

In the present study, we investigated the role of individual and contextual factors on inter-limb asymmetry in postural control. Three major findings emerged, i. e., inter-limb performance differences a) were lower in athletes with (i. e., soccer players) than without (i. e., swimmers) the preferential use of one leg for postural control; b) did not differ between soccer players with diverging levels of training experience (i. e., 2–5 or 6–9 years); and c) occurred in soccer players during ecological but not non-ecological test conditions.

### Inter-limb asymmetry in postural control: role of individual factors

The first assumption that inter-limb asymmetry is greater in athletes with (i. e., soccer players) than without (i. e., swimmers) the preferential use of one leg for postural control and age-matched non-athletes was not confirmed and is in contrast to previous studies 13 15 . Conversely, soccer players (LSI=5.4%) compared to swimmers (LSI=9.2%) even showed significantly less inter-limb performance differences. One possible explanation could be that unilateral actions such as passing, crossing, and kicking often occur in soccer matches, but are routinely practiced during training with both the dominant and non-dominant limb 26 . Bilateral practice enhances the technical repertoire and provides more opportunities to be successful in one-to-one scenarios. Moreover, practicing with both limbs prevents muscular imbalances 27 and may reduce the risk of injury. In addition to the soccer-specific training, athletic training is also carried out, consisting of endurance, strength, sprinting, and agility exercises 28 , most of which are performed on both legs. This could also have counteracted the development of inter-limb asymmetry in soccer players.

Overall, our findings indicate that the repeated long-term practice of unilateral soccer-specific actions such as passing, crossing, and kicking does not have a detrimental effect in terms of an increased level of inter-limb asymmetry in soccer players, but seems to be compensated for by bilateral exercises (running, sprinting, and change-of-direction drills) that also take place during training and competition 29 30 .

The significantly greater asymmetry values of swimmers compared to soccer players are contrary to our expectations and can be explained by the fact that the former show a foot posture with a tendency towards pronation, and a Q-angle with a tendency towards valgus, while for the latter the foot posture is within the normal range 31 . These biomechanical discrepancies in swimmers compared to soccer players can favor the development of asymmetries and thus negatively influence postural control 32 . In this regard, Matsuda et al. 33 showed lower postural sway in the AP and ML directions during the single leg stance in soccer players compared to swimmers.

In contrast to the second hypothesis, which states that inter-limb asymmetry is more pronounced in soccer players with a high (i. e., 6–9 years) compared to a low (i. e., 2–5 years) level of training experience, no significant group differences were found. This finding is in accordance with the results of Leinen et al. 12 , who also reported no inter-limb differences in balance performance between soccer players with diverging levels of training experience (i. e., U13, U15, and U19) but contradicts other studies 9 34 35 that have shown greater inter-limb performance differences in more compared to less experienced players. For example, Muehlbauer et al. 35 compared reach distances for the YBT–LQ between limbs in young soccer players and detected an increase in side-to-side differences for the AT reach direction from U13 over U15 and U17 to U19 players. Differences in the amount of training experience could be a possible explanation. Specifically, in the present study, training experience of 2–5 years and 6–9 years was less compared to the work of Muehlbauer and colleagues 35 , which ranged on average from seven to ten years and may not adequate to induce structural and functional adaptations in the postural control system 13 15 16 . In addition, the accumulation of the above-mentioned bilateral practice increases with increasing training experience and thus making an increase of inter-limb asymmetry less likely.

### Inter-limb asymmetry in postural control: role of contextual factors

Consistent with our third hypothesis and previous literature 15 16 , inter-limb performance differences in soccer players were detected during ecological (i. e., single-leg drop landing task) but not non-ecological (i. e., YBT–LQ) test conditions. Although both tasks represent dynamic balance, single leg reaching movements in different directions are rarely found in soccer and are therefore not ecologically valid. In contrast, single leg landings are a more common part of training (e. g., plyometric jumps) and competition (i. e., heading movements following a corner) 36 and can therefore be categorized as ecologically valid. The observation of greater differences under ecological rather than non-ecological test conditions can be explained by the fact that, depending on the type of sport, specific postural adaptations occur with regard to the perception, transfer, and processing of visual, vestibular, and somatosensory information and consequently lead to enhanced postural skills 15 . From a practical perspective, our finding suggest that ecological test conditions are more appropriate for investigating the development of inter-limb asymmetry in soccer players.

The findings of the present study should be interpreted in light of some limitations. Firstly, we performed a cross-sectional study which does not allow drawing cause-and-effect relationships. Secondly, task specificity represents only one contextual factor. The investigation of additional factors such as task difficulty by means of a gradual reduction of the base of support (e. g., from bipedal over tandem to unipedal stance), sensory manipulations (e. g., standing with eyes open/closed on firm/foam ground), or the concurrent execution of a cognitive task (e. g., decision-making) would be quite valuable for future work. Thirdly, inter-limb performance differences were determined on a behavioral but not on a neuromuscular level. Thus, future work should additionally examine muscle activity during balance assessment to expand our findings.

## Conclusions

This work provides additional insights into the role of individual and contextual factors on inter-limb asymmetry in postural control. With respect to individual factors, we found that inter-limb performance differences were lower in athletes with (i. e., soccer players) than without (i. e., swimmers) the preferential use of one leg for postural control. This indicates that the predominant long-term use of one limb for balance requirements does not necessarily lead to a higher magnitude of inter-limb asymmetry. Even with increased training experience, no significant differences in inter-limb asymmetry could be observed in soccer players. However, with respect to contextual factors (i. e., balance task specificity), inter-limb asymmetry was detected under ecological test conditions. This finding indicates the development of sport-specific postural skills. From a practical perspective, it might be advisable to create contextualized test conditions related to the practiced type of sport in order to increase the probability of detecting inter-limb performance differences.

## Funding Information

Deutsche Forschungsgemeinschaft — http://dx.doi.org/10.13039/501100001659; MU 3327/5-1 and PA 774/21-1
